# Supplementary material for: Efficacy of colchicine in patients with moderate COVID-19: A double-blinded, randomized, placebo-controlled trial
Source: PLoS One. 2022 Nov 16;17(11):e0277790. doi: 10.1371/journal.pone.0277790 (PMC9668149; doi:10.1371/journal.pone.0277790)
Supplement: S1 Fig — (DOCX) [file pone.0277790.s002.docx]

Supplementary Fig 1: Forest plot: showing sub group effect at day 14
