# Supplementary material for: Efficacy of colchicine in patients with moderate COVID-19: A double-blinded, randomized, placebo-controlled trial
Source: PLoS One. 2022 Nov 16;17(11):e0277790. doi: 10.1371/journal.pone.0277790 (PMC9668149; doi:10.1371/journal.pone.0277790)
Supplement: S1 Table — (DOCX) [file pone.0277790.s003.docx]

|  | Colchicine | | | Placebo | | | | |
| --- | --- | --- | --- | --- | --- | --- | --- | --- |
| Day - 1 vs. Day - 7 | | | | | | | | |
| Biomarkers Parameters | Day - 1  Median (IQR)(n) | Day - 7  Median (IQR)(n) | p-value | | Day - 1  Median (IQR)(n) | Day - 7  Median (IQR)(n) | p-value | |
| Serum CRP | 10.0 (6.0-28.2) (n=148) | 6.0 (5.6-12.0) (n=55) | <0.01 | | 15.2(6.0-25.0)(n=148) | 6.0(6.0-20.6) (n=56) | 0.01 | |
| Serum Ferritin | 301.0 (175.0-758.0) (n=148) | 365.0 (213.5-613.0) (n=61) | 0.83 | | 256.0(95.0-642.0)(n=148) | 300.0 (170.2-667.2)(n=54) | 0.29 | |
| D- dimer | 0.4 (0.3-0.8) (n=148) | 0.4 (0.3-0.9) (n=59) | 0.84 | | 0.5(0.3-1.5)(n=148) | 0.4(0.3-1.1)(n=52) | 0.83 | |
| Day - 1 vs. Day - 14 | | | | | | | | |
| Serum CRP | 10.0 (6.0-28.2) (n=148) | 3.0 (1.2-6.4) (n=20) | 0.02 | | 15.2(6.0-25.0)(n=148) | 6.0(1.8-13.5) (n=15) | | 0.01 |
| Serum Ferritin | 301.0 (175.0-758.0) (n=148) | 331.2 (219.5-798.6) (n=20) | 0.48 | | 256.0(95.0-642.0)(n=148) | 365.0(140.0-530.0)(n=19) | | 0.88 |
| D- dimer | 0.4 (0.3-0.8) (n=148) | 0.4 (0.1-0.8) (n=14) | 0.27 | | 0.5(0.3-1.5)(n=148) | 0.3(0.1-1.5)(n=14) | | 0.21 |

Wilcoxon signed rank test-related sample

Supplementary Table 1: Comparison of baseline and follow up blood biomarkers between Colchicine and Placebo
