# Supplementary material for: Efficacy of colchicine in patients with moderate COVID-19: A double-blinded, randomized, placebo-controlled trial
Source: PLoS One. 2022 Nov 16;17(11):e0277790. doi: 10.1371/journal.pone.0277790 (PMC9668149; doi:10.1371/journal.pone.0277790)
Supplement: S2 Table — (DOCX) [file pone.0277790.s004.docx]

| Outcomes | Colchicine (n=146) | Placebo (n=146) | Odds Ratio  or Hazard Ratio  (95% CI) | p-value |
| --- | --- | --- | --- | --- |
| Deterioration of 2 points in the ordinal outcome within 14 days— no. of patients (%) | 4 (2.7) | 9 (6.2) | Hazard Ratio;  0.44 (0.13 - 1.43) | 0.171 |
| Clinical status at 14 days — no. of patients (%) |  |  |  |  |
| Home with no restriction | 82 (56.2) | 82 (56.2) |  |  |
| Home with some restriction | 18 (12.3) | 16 (11.0) |  |  |
| Hospitalized without supplemental oxygen requirement | 37 (25.3) | 36 (24.7) |  |  |
| Hospitalized with Mask Nasal cannula oxygen | 5 (3.4) | 3 (2.0) |  |  |
| Hospitalized with Non-invasive Ventilation HFNC | 2 (1.4) | 4 (2.7) |  |  |
| Hospitalized with Invasive ventilation | 0 (0.0) | 0 (0.0) |  |  |
| Death | 2 (1.4) | 5 (3.4) |  |  |
| Length of hospital stay after enrolment (Mean±SD) | 11.43±7.495 | 11.24±7.482 |  | 0.829 |

Supplementary Table 2: clinical outcome at day 14.
