# Supplementary material for: Efficacy of colchicine in patients with moderate COVID-19: A double-blinded, randomized, placebo-controlled trial
Source: PLoS One. 2022 Nov 16;17(11):e0277790. doi: 10.1371/journal.pone.0277790 (PMC9668149; doi:10.1371/journal.pone.0277790)
Supplement: S3 Table — (DOCX) [file pone.0277790.s005.docx]

| Clinical outcomes at day 28 | | |
| --- | --- | --- |
| Outcomes | **Colchicine (N=146)** | **Placebo (N=146)** |
|  | n (%) | n (%) |
| Home with no restriction (1) | 134 (91.8) | 126 (86.3) |
| Home with some restriction (2) | 3(2.1) | 5 (3.4) |
| Hospitalized without supplemental oxygen requirement (3) | 2(1.4) | 2 (1.4) |
| Hospitalized with Mask Nasal cannula oxygen (4) | 3(2. 1) | 0 (0.0) |
| Hospitalized with Non-invasive Ventilation HFNC (5) | 0(0.) | 0 (0.0) |
| Hospitalized with Invasive ventilation (6) | 0 (0.0) | 0 (0.0) |
| Death (7) | 4(2.7) | 13(8.9) |

Supplementary Table 3: clinical outcome at day 28.
