# Supplementary material for: Efficacy of colchicine in patients with moderate COVID-19: A double-blinded, randomized, placebo-controlled trial
Source: PLoS One. 2022 Nov 16;17(11):e0277790. doi: 10.1371/journal.pone.0277790 (PMC9668149; doi:10.1371/journal.pone.0277790)
Supplement: S1 Protocol — (DOCX) [file pone.0277790.s006.docx]

**Title: Colchicine in moderate symptomatic COVID-19 patients: double blind, randomized, placebo controlled trial to observe the efficacy**

**Principal investigator:**

**Dr. Motlabur Rahman**

Associate professor

Department of Medicine

Dhaka Medical College, Dhaka.

**Table of Content**

| **Topic** | **Page no.** |
| --- | --- |
| Abbreviations | **3** |
| Protocol summary | **4** |
| Introduction | **6** |
| Background | **6** |
| Information about study drug | **8** |
| Hypothesis | **9** |
| Rationale | **9** |
| Objectives and outcomes | **10** |
| Study site | **10** |
| Methodology, factors in study | **11** |
| Study design | **12** |
| study population | **13** |
| Selection criteria | **13** |
| Strategies for Recruitment and Retention | **13** |
| Participant timeline | **14** |
| Sampling | **15** |
| Sample size with statistical basis | **15** |
| Procedure of intervention | **16** |
| Assignment of interventions | **17** |
| Methods of Data Collection | **18** |
| Study activities | **18** |
| Pretesting | **19** |
| Statistical analysis | **19** |
| Patient Safety Monitoring | **20** |
| Study intervention discontinuation and participation withdrawal | **21** |
| Ethical considerations | **21** |
| Budget and funding | **21** |
| Supporting documents and operational considerations | **21** |
| References | **22** |
| Appendix 1 | **23** |
| Inform written consent form ( Bengali) | **25** |
| Inform written consent form (English) | **27** |
| Data collection sheet | **29** |
| Screening for enrollment sheet | **34** |

**Abbreviations**

| CS | cytokine storm |
| --- | --- |
| CAP | Community Acquired Pneumonia |
| CBC | Complete Blood Count |
| COVID-19 | Coronavirus Disease 2019 |
| CP | Convalescent Plasma |
| CRP | C-Reactive Protein |
| ICU | Intensive Care Unit |
| ILI | Influenza like illness |
| DMCH | Dhaka medical college hospital |
| LFT | Liver Function Tests |
| MERS-CoV | Middle eastern respiratory syndrome |
| RT-PCR | Real time- Polymerase Chain Reaction |
| WHO | World health organizations |
| SARS | Severe acute Respiratory Syndrome |
| SARS-CoV-2 | Severe Acute Respiratory Syndrome Coronavirus 2 |

**PROTOCOL SUMMARY**

| **Title:** | **Colchicine in moderate symptomatic COVID-19 patients: double blind, randomized, placebo controlled trial to observe the efficacy** |
| --- | --- |
| **Principal investigator:** | Dr. Motlabur Rahman, Associate professor, Department of Medicine, Dhaka Medical College, Dhaka. |
| **Study Description:** | This is a prospective, double blind, randomized, placebo controlled clinical trial. We will randomize the Patients into two groups (group A and group B). Patients of group-A are the treatment group. They will be treated with optimal treatment based on the algorithm proposed in National Guidelines on Clinical Management of Coronavirus Disease 2019 (Covid-19) Version 7.0, 28 May 2020, along with Colchicine for 14 days. The patients in group-B will be controlled group. They will be treated with optimal treatment based on the algorithm proposed in National Guideline along with a placebo. |
| **Objectives:** | **Primary:**  To see the efficacy of Colchicine in moderate symptomatic COVID-19 patients  **Secondary:**  To see-  Clinical deterioration of the patients  Need for supplemental oxygen  The need for non-invasive or invasive ventilation  In hospital mortality |
| **Outcomes:** | **Primary:**  Outcome Name: Time to develop clinical deterioration, defined as the time from randomization to a deterioration of two points (from the status at randomization) on a Seven-category ordinal scale.  Metric/method of measurement: Seven-category ordinal scale.  Time point: 14 days following randomization  **Secondary:**  1.Length of hospital stay  Time frame: 14 days post randomization  2.Number of participant requiring increased amount of supplemental oxygen  Time frame: 14 days post randomization  3.Number of participants requiring mechanical ventilation  Time frame: 14 days post randomization  4.Number of participants who die  Time frame: 14 days post randomization |
| **Study Population:** | A patient with laboratory confirmation of COVID-19 infection with clinical signs and symptoms of moderate disease |
| **Sample size:** | Sample size in colchicine group-150  Sample size in control group- 150 |
| **Study site:** | Dhaka Medical College Hospital, Dhaka, Bangladesh. |
| **Description of Study Intervention:** | Following signature of the informed consent form, 300 subjects meeting all inclusion and having no exclusion criteria will be randomized to receive either group A or group B (1:1 allocation ratio) for 14 days. All patients will be classified according to seven-category ordinal scale. Each patient will get treatment according to its group e.g group A or group B.  Colchicine arm: People in the colchine group will be given a starting dose of 1.2 mg (2 tablets of 0.6 mg )single or 12 hourly divided dose. After that, they will take colchicine 0.6mg daily for 13 days. If they develop gastro intestinal side effects e.g abdominal pain, burning, vomiting, diarrhea, omeprazole and antiemetic will be prescribed. Supportive care and treatment will also be given.  Placebo arm: COVID-19 Patients in this arm will receive standard COVID-19 treatment according to national guidelines of Bangladesh and will receive placebo.  Standard care of enrolled study patients will consist:  1.Isolation facility  2.Symptomatic treatment with Paracetamol, Fexofenadine  3. Steam inhalation/Gurgle of Lukewarm water.  4. Ensuring of hand wash (20 seconds each time) and ideally wearing mask.  5. Monitoring by the attending nurses. |
| **Study Duration:** | 6 months |

**Introduction:**

A deadly pandemic of COVID- 19 has been started from Wuhan, china during early December, 2019. So far 215 Countries, areas or territories already involved throughout the glove leaving 5934936 patients infected and 367166 killed. Bangladesh is also affected since it’s first case detection on 8^th^ April, 2020. Up to May 31, 2020 the total number of positive case was 44608 with 610 deaths [1]. As the SARS-CoV-2 making its way around the world, researchers and doctors are in search for drugs to treat and stop the spread of the disease. Since, there are no specific therapeutic options available at present, health officials are primarily relying on quarantining the infected to contain the virus spread and repurposing already existing anti-viral drugs and antibiotics to treat infection based on symptoms.

**Background:**

The disease caused by novel SARS-CoV-2 is named as COVID-19 by World Health Organization (WHO). The culprit virus belongs to the Coronaviridea family of corona viruses that caused two other outbreaks, namely Severe Acute Respiratory Syndrome (SARS) in 2002 and Middle East Respiratory Syndrome (MERS) in 2012.

There are at least 39 corona viruses, among which seven are most commonly found infecting human:

1. 229E (alpha coronavirus)

2. NL63 (alpha coronavirus)

3. OC43 (beta coronavirus)

4. HKU1 (beta coronavirus)

5. MERS-CoV (the beta corona virus that causes Middle East Respiratory Syndrome or MERS)

6. SARS-CoV (the beta corona virus that causes severe acute respiratory syndrome or SARS)

7. SARS-CoV-2 (the novel coronavirus that causes coronavirus disease 2019, or COVID-19).

SARS-CoV-2 is a single stranded, positive strain RNA virus with a protein shell and membrane. Due to their dependency on hosts’ molecular machinery to replicate themselves, these RNA based viruses are prone to mutations when they transfer from one animal to another. When infection transfers from animal to human, they can evolve into new virus like SARS-CoV, MERS-CoV and SARS-CoV-2. Genetic information of the virus is the starting point in understanding the origin of the pathogen and one of the valuable information in designing strategies to fight against it. As soon as the genetic makeup of SARS-CoV-2 is revealed by Li-Li Renet.al on January 25th 2020, researchers across globe began comparing the novel coronavirus’s genome with SARS and MERS to determine if any of the drugs developed against SARS and MERS can work against this novel SARS-CoV-2. [2].

Based on ongoing epidemiological investigations, the incubation period of the virus is 1-14 days, mostly
3-7 days. SARS-CoV-2 is contagious during the latency period. Based on the knowledge acquired
during these months the infection has been divided into three phases, the first asymptomatic or with
mild symptoms, the second and third characterized by hyperactive inflammatory state responsible for
lung lesions and that in some patients can rapidly develop acute respiratory distress syndrome, respiratory failure, multiple organ failure, and even death. Phase two and three of the infection is characterized by a hyperactive inflammatory state that can result in a cytokinic storm (CS) [3]. Sudden and rapidly progressing clinical deterioration has been widely mentioned in late stages of COVID-19 (around 7-10 days). This often manifests as an unexpected aggravation of symptoms (fever,
dyspnea) and is correlated with increased levels of acute phase reactants (ESR, CRP, ferritin), coagulopathy (elevated titers of d-dimers, disseminated intravascular coagulation), and cell lysis (CK, LDH).In the most severe patients, clinical and laboratory parameters correlated with increased levels of proinﬂammatory cytokines (IL-1β, IL-1Ra, IL-6, TNF-α, and sIL2-Rα), evocative of a cytokine storm. Interestingly, ARDS occurs in SARS-CoV patients despite a diminishing viral load, suggesting that exuberant host immune response may be responsible for this outcome rather than viral virulence. Such a cytokine profle is strongly reminiscent of both Cytokine Release Syndrome (CRS, seen in CAR T cell therapy) and hemophagocytic lymphohistiocytosis (HLH). Numerous authors have paralleled the COVID-19 cytokine storm to either primary or reactive HLH (reHLH) because of its close resemblance, including high fever, cytopenia, hyperferritinemia, abnormal liver tests, coagulopathy, and pulmonary involvement (including ARDS), occurring in approximately 50% of patients with reHLH. In adults, reHLH is most often triggered by viral infections and is observed in 3 - 4% of sepsis cases. Herpesviridae (e.g. Epstein-Barr virus) and inﬂuenza are major triggers of such cytokine storms. Systemic diseases, like systemic lupus erythematosus, or the autoinﬂammatory adult-onset Still’s disease and its pediatric counterpart, can also be complicated by cytokine storm, known as the macrophage activation syndrome (identical to reHLH). In all these conditions, IL-1β, IL-18, IFN-γ, and IL-6 are key mediators of hyperinﬂammation. Analysing more precisely the immunopathology of SARS-CoV2-related ARDS, Giamarellos-Bourboulis *et al.* have come to the conclusion that two patterns of immune dysfunction exist in worsening COVID-19: (i) one pattern highly suggestive of macrophage activation syndrome (hyperferritinemia and elevated H score : 25% of patients), which is driven by IL-1β; and (ii) one pattern with immune dysregulation driven by IL-6 [71]. The latter was characterized by a combination of hypercytokinemia, immunoparalysis (as indicated by decreased HLA-DR molecules on CD14 monocytes), and global lymphopenia (including CD4+ and NK cells). Interestingly, IL-6 blockade with tocilizumab partially restored HLA-DR expression on CD14 monocytes and increased the circulating lymphocyte coun[4].

Based on the inflammatory nature of the severe COVID-19 disease many anti-inflammatory drugs have been studied with variable efficacy. They includes targeted anti inflammatory therapy e.g IL 1 , IL6 inhibitors, interferon gamma,TNF alfa inhibitors, and non targeted anti inflammatory e.g corticosreroid, hydroxychloroquine, JAK inhibitors and colchicine. Among them Colchicine is a long-established drug with anti-inflammatory properties used to treat patients with Behçet’s disease or familial Mediterranean fever. Colchicine inhibits IL-1β and its subsequent inflammatory cascade principally by blocking pyrin and (to a lesser ex-tent) NLRP3 inflammasome activation. Until now, there has been no data indicating that pyrin is activated upon SARS-CoV-2 infection and this would appear rather unlikely. On the other hand, NLRP3 is likely to be activated following virus entry into the cell. Nevertheless, the inhibition of NLRP3 inflammasome by colchicine has not been firmly demonstrated since in vitro it does not prevent IL 1βsecretion induced by typical NLRP3 stimuli (i.e.ATP,nigericin). How ever, five RCTs are currently under way to test the efficacy of colchicine in COVID-19 patients (NCT04322682, NCT04322565, NCT04328480,NCT04326790, NCT04350320 [4].

**Information about study drug:**

Two major biologic effects of colchicine are well known: its ability to arrest mitosis in metaphase and its clinical anti-inflammatory effect in the treatment of acute gout.

Indications:

Acute gout; short-term prophylaxis during initial therapy with allopurinol and uricosuric drugs; prophylaxis of familial Mediterranean fever (recurrent polyserositis)

Cautions:

In elderly; gastro-intestinal disease; cardiac disease;

Interactions:

Azithromycin-possible increased risk of colchicine toxicity when given with azithromycin —suspend or reduce dose of colchicine (avoid concomitant use in hepatic or renal impairment)

Contra-indications:

blood disorders, Hepatic impairment, use with caution Renal impairment -reduce dose or increase dosage interval if eGFR 10–50 mL/minute/1.73 m^2^; avoid if eGFR less than 10 mL/minute/1.73 m^2^, Pregnancy-avoid—teratogenicity in *animal* studie, Breast-feeding - present in milk but no adverse effects reported; manufacturers advise caution

Side-effects:

Nausea, vomiting, and abdominal pain; excessive doses may cause profuse diarrhoea, gastro-intestinal haemorrhage, rash, renal and hepatic damage; *rarely* peripheral neuritis, inhibition of spermatogenesis, myopathy, alopecia, and with prolonged treatment blood disorders

Dose

- Acute gout, 500 micrograms 2–4 times daily until symptoms relieved, max. 6 mg per course; course not to be repeated within 3 days
- Prevention of gout attacks during initial treatment with allopurinol or uricosuric drugs, 500 micrograms twice daily
- Prophylaxis of familial Mediterranean fever, 0.5–2 mg once daily

(Source-British National Formulary)

**Hypothesis:**

Based on preliminary evidence, that the anti-inflammatory effects of colchicine may prevent or minimize the cytokine storm and limit the organ damage. Thus it will prevent clinical deterioration, hospitalization, shift to ICU and reduce mortality. So, our hypothesis is-

1. Colchicine is effective in the treatment of moderate COVID-19 patient than placebo.

**Rationale:**

In Bangladesh colchicine is a readily available drug, has been used for many years for established indications. it is a cheap drug. Specific targeted anti-inflammatory drugs like Tocilizuma, Anakinra is costly. As a low economic country these drugs will not be affordable to our people as well as it will be a burden for the government. If colchicine is found to be effective in the treatment of COVID-19 it can be recommended in our national guidelines.

**Objectives and outcomes:**

**Study objectives:**

**Primary:**

To see the efficacy of Colchicine in moderate symptomatic COVID-19 patients

**Secondary:**

To see-

Clinical deterioration of the patients

Need of supplemental oxygen

The need for non-invasive or invasive ventilation

In hospital mortality

**Study Outcomes:**

**Primary:**

Outcome Name: Time to develop clinical deterioration, defined as the time from randomization to a deterioration of two points (from the status at randomization) on a Seven-category ordinal scale.

Metric/method of measurement: Seven-category ordinal scale. The scale is recommended by the WHO R&D Blueprint expert group. The seven-category ordinal scale consisted of the following categories: 1, not hospitalized with resumption of normal activities; 2, not hospitalized, but unable to resume normal activities; 3, hospitalized, not requiring supplemental oxygen; 4, hospitalized, requiring supplemental oxygen; 5, hospitalized, requiring nasal high-flow oxygen therapy, noninvasive mechanical ventilation, or both; 6, hospitalized, requiring ECMO, invasive mechanical ventilation, or both; and 7, death [5][6][7].

Time point: 14 days following randomization

**Secondary:**

1.Length of hospital stay

Time frame: 14 days post randomization

2.Number of participant requiring increased amount of supplemental oxygen

Time frame: 14 days post randomization

3.Number of participants requiring mechanical ventilation

Time frame: 14 days post randomization

4.Number of participants who die

Time frame: 14 days post randomization

**Study site:** Dhaka Medical College Hospital, Dhaka, Bangladesh.

**Methodology:**

**Factors in study (variables):**

1. Age and sex of patient
2. Residential area of patient.
3. Profession:
4. Symptoms- fever, cough, sore throat, Shortness of breath, diarrhea, body ache
5. Clinical stage – On WHO seven-category ordinal scale
6. Data about various treatments including:
7. Anti pyretics
8. Antiviral therapies and
9. Steroids
10. others
11. Clinical Data including:
12. Body temperature
13. O_2_ Saturations
14. Laboratory data including
15. Complete Blood Count
16. Screening tests- RBS, ALT, S .Creatinine
17. RT-PCR
18. Inflammatory factors C-reactive protein (CRP)
19. Data from chest imaging (chest x ray and/or CT scan of chest) and ECG
20. Information on complication:

**Study design:**

This is a prospective, double blind, randomized, placebo controlled clinical trial. We will randomize the Patients into two groups (group A and group B). Patients of group-A are the treatment group. They will be treated with optimal treatment based on the algorithm proposed in National Guidelines on Clinical Management of Coronavirus Disease 2019 (Covid-19) Version 7.0, 28 May 2020, Disease Control Division, Directorate General of Health Services, Ministry of Health & Family Welfare, Government of the People's Republic of Bangladesh along with Colchicine [8]. The patients in group-B will be controlled group. They will be treated with optimal treatment based on the algorithm proposed in National Guideline along with a placebo. Following is the study design in short-

| Study Type : | Interventional  (Clinical Trial) |
| --- | --- |
| Proposed Enrollment : | 300 participants |
| Sample Allocation: | Randomized |
| Intervention Model: | Parallel Assignment |
| Masking: | Double (participant and investigator) |
| Comparator:  Primary Purpose: | placebo  Treatment and prevention of the development of severe disease. |
| Study Title: | Colchicine in moderate symptomatic COVID-19 patients: double blind, randomized, placebo controlled trial to observe the efficacy |
| Proposed Study Start Date : | June , 2020 |
| Proposed Study Duration : | 6 months from start date |

**Study Population:**

A patient with laboratory confirmation of COVID-19 infection with clinical signs and symptoms of moderate disease

**Selection criteria:**

**Inclusion criteria:**

1. Males and females of least 18 years of age and can swallow tablets
2. Competent and willing to provide informed consent
3. Patient must have received a diagnosis of COVID-19 infection with positive RT-PCR for SARS CoV-2 within the last 3 days
4. Patients with moderate symptoms (According to National Guidelines on Clinical Management of Coronavirus Disease 2019 (COVID- 19).Version 7.0 .2020, DGHS, MOHFW, Government of the People's Republic of Bangladesh)(Appendix 1)

**Exclusion criteria:**

1. Pregnancy and breast-feeding
2. Known hypersensitivity to colchicine
3. Known chronic illness e.g hepatic failure, chronic kidney disease (eGFR<30ml/min), decompensated heart failure, long QT syndrome (QTc >450 msec.)
4. Patient with inflammatory bowel disease (Crohn's disease or ulcerative colitis), chronic diarrhea or malabsorption
5. Patient currently taking colchicine for other indications (mainly chronic indications represented by Familial Mediterranean Fever or gout)
6. Patient undergoing chemotherapy for cancer
7. Patient is considered by the investigator, for any reason, to be an unsuitable candidate for the study.

**Strategies for Recruitment and Retention:**

All RT-PCR positive patients admitted in DMCH is our study population. From them sample will be collected based on inclusion and exclusion criteria. Patients will be properly informed and written consent will be taken. We will randomize the Patients into two groups (group A and group B). Intervention will be done according to protocol. Patients will preserve the right to discontinue the trial at any time and this will not influence his standard care.

**Participant timeline**

*Table.1: Study timeline of participant assessments/interventions*

|  | **STUDY PERIOD** | | | | | | | |
| --- | --- | --- | --- | --- | --- | --- | --- | --- |
|  | **Screening/baseline/**  **randomization** | **Treatment phase** | **Follow-up phase** | | | | | **Data processing and analysis** |
| **TIMEPOINT** | **D0** | **D1–D14** | **D3** | **D7** | ***D10*** | ***D14*** | ***D 21*** | ***Up to 6 Months*** |
| **ENROLMENT:** |  |  |  |  |  |  |  |  |
| Inclusion/exclusion criteria | X |  |  |  |  |  |  |  |
| Written informed consent | X |  |  |  |  |  |  |  |
| Demographics | X |  |  |  |  |  |  |  |
| Assessment of concomitant chronic diseases | X |  |  |  |  |  |  |  |
| Efficacy/safety assessment | X | X | X | X | X | X | X |  |
| ECG | X |  |  |  |  |  |  |  |
| Chest imaging | X |  |  |  |  |  |  |  |
| Vital signs | X | X |  |  |  |  |  |  |
| (body temperature, oxygen saturation) | X | X | X | X | X | X |  |  |
| Clinical symptoms assessment | X |  |  |  |  |  |  |  |
| Urine for pregnancy test | X |  |  |  |  |  |  |  |
| (fever, cough, diarrhea, dyspnea) | X | X | X | X | X | X | X |  |
| **INTERVENTIONS:** |  |  |  |  |  |  |  |  |
| Colchicine/placebo |  | X |  |  |  |  |  |  |
| Concomitant medications assessment |  |  | X | X | X | X |  |  |
| **LAB ASSESSMENTS:** |  |  |  |  |  |  |  |  |
| Whole blood count (sampling time) | X |  | X | X | X | X |  |  |
| Serum Creatinine, SGPT, RBS | X |  |  | X |  | X |  |  |
| CRP, Serum Ferritin, d-Dimer, LDH |  |  | X | X | X | X |  |  |
| Nasopharyngeal swab for RT-PCR for SARS-CoV-2, (up to 2 negative) |  |  | X | X | X | X |  |  |

**Sampling:**

Simple random sampling

**Sample size with statistical basis:**

This double blinded, placebo controlled trial proposes to assess the effectiveness of colchicine in curing patients suffering from COVID19 in comparison to placebo. A previous study showed that proportion of subjects cured by colchicine is 50% and a clinically important difference of 16% as compared to placebo is acceptable.

Level of significance = 5%, Power = 80%, Type of test = two-sided

Formula of calculating sample size is [9].

n = [(Z_α/2_ + Z_β_)^2^ × {(p1 (1-p1) + (p2 (1-p2))}]/(p1 - p2)^2^

where

n = sample size required in each group,

p1 = proportion of subject cured by colchicine = 0.50,

p2 = proportion of subject cured by placebo = 0.34,

p1-p2 = clinically significant difference = 0.16

Z_α/2_: This depends on level of significance, for 5% this is 1.96

Z_β_: This depends on power, for 80% this is 0.84

Based on above formula the sample size required per group is 146. Hence total sample size required is 292.

So, a sample size of 292 patients, 146 in each arm, is sufficient to detect a clinically important difference of 16% between groups in curing COVID19 using a two-tailed z-test of proportions between two groups with 80% power and a 5% level of significance. This 16% difference represents a 50% cure rate using colchicine and 34% cure rate using standard treatment and placebo.

For the purpose of convenience we will recruit total of 300 patients, 150 patients in colchicine treatment group and 150 patient in control group ( placebo group). So,

Sample size in colchicine group-150

Sample size in control group- 150

**Procedure of intervention:**

This will be a randomized, prospective, double blinded, placebo controlled, single-center study. Following signature of the informed consent form, 300 participants meeting all inclusion and having no exclusion criteria will be randomized to either treatment group or placebo group (1:1 allocation ratio) for 14 days. All patients will be in category 3, hospitalized, not requiring supplemental oxygen; according to seven-category ordinal scale. Patients with other categories will not be included in this study. Each patient will get treatment according to its group e.g group A or group B.

| **Arm** | **Interventions** |
| --- | --- |
| **Group A ( TRAETMENT GROUP)** | People in the colchine group will be given a starting dose of 1.2 mg (2 tablets of 0.6 mg )single or 12 hourly divided dose. After that, they will take colchicine 0.6mg daily for 13 days. If they develop gastro intestinal side effects e.g abdominal pain, burning, vomiting, diarrhea, omeprazole and antiemetic will be prescribed. Supportive care and treatment will also be given. |
| **Group B ( CONTROL (PLACEBO) GROUP)** | COVID-19 Patients in this arm will receive standard COVID-19 treatment according to national guidelines of Bangladesh and will receive placebo.    Standard care of enrolled study patients will consist:  1.Isolation facility  2.Symptomatic treatment with Paracetamol, Fexofenadine  3. Steam inhalation/Gurgle of Lukewarm water.  4. Ensuring of hand wash (20 seconds each time) and ideally wearing mask.  5. Monitoring by the attending nurses. |

**Assignment of interventions: allocation**

Sequence generation

Three hundred identical opaque envelopes will be prepared by an independent statistician. He will mark them from 1 to 300. Each envelope will contain either a full course of drugs or placebo for one patient. Envelops will be filled randomly and statistician will keep a record of its content in a secured place. Colchicine and placebo blisters within each envelope are identical in size and labeling. The statistician will supply 300 identical cards containing numbers 1 to 300. After signing informed consent each participant will take a card randomly by lottery. That number will be assigned to the participant as his ID number. The study nurse will supply the sealed envelope with the same number containing either drug or placebo.

Concealment mechanism

Identical opaque sealed envelopes will be prepared by an independent statistician. The information about the content of each envelop will be kept secured by the statistician. No investigator or study personnel will have access to that. Colchicine and placebo tablets will have the same size, shape, colour and smell. The Colour, size, shape of the blister and the labeling will be the same. Investigators will know the number of the envelope but not the content.

**Assignment of interventions: Blinding**

Who will be blinded?

This study is a double-blind clinical trial. The study participants, investigators, care providers, outcome assessors, and data analysts are all blinded. Treatment allocation will only be decoded after complete data analysis.

Procedure for unblinding if needed

Unblinding is permissible when investigators believe that there is a very strong need to know the study drug allocation to perform any specific treatment/ action for the safety of the participant. Whenever possible, unblinding will only be conducted after discussion with the study principal investigator. Unblinding and the situation lead to it will be reported to the ethical review committee. The procedure for revealing a participant's allocated intervention is as follows: (1) investigators confirm that the patient meets the criteria of unblinding according to the protocol; (2) the independent physician not related to the study will open the sealed envelope and inform the investigators of the allocation; (3) information about the date, time, and reason of unblinding will be recorded in the envelope; (4) the envelope must be sealed again as soon as possible and securely stored.

**Methods of Data Collection:**

Data will be collected from the subjects using a preformed questionnaire. Hospitalized patients will be interviewed by one of the investigators on day 1 and will record information in questionnaire. Day of randomization and day of starting treatment will be the same and this will be marked as day 1. Daily follow up will be given by treating physician and inform investigator if needed.one of the Investigators will follow up the participant on day 3, day 7, day 10, day 14 and day 21 and will record data . Patients who are at home on day 21 will be followed up over phone call or video call by investigators. All data will be recorded in data collection form.

**Study activities:**

Screening visit:

Obtaining informed written consent

Selection of sample according to inclusion and exclusion criteria

Obtaining history and physical examination

Blood sample for screening investigations- CBC, Creatinine, SGPT, RBS

Urine for pregnancy test in female of childbearing age

Chest x-ray and/ or CT scan of chest, ECG

Day of randomization/ Day 1:

Randomization to either group A or group B

RT- PCR from nasopharyngeal swab, if not already done

CRP, Serum Ferritin

Initiation of study drug or placebo

Follow up

Day 2 to day 14 visits:

New complaints

Physical examinations

Oxygen saturation

CBC, Creatinine, SGPT, RBS, Chest X-ray, ECG on day 4

RT-PCR for SARS-CoV-2 on day 4, day 7 and day 14 or on the day of discharge( Up to 2 consecutive test negative)

Continuation of study drug or placebo

Follow up on day 21:

From history

**Pretesting:**

Pretesting will be performed to find out the feasibility of the study.

**Statistical methods**

Statistical methods for primary and secondary outcomes

The primary efficacy analysis will be performed on an intention-to-treat basis. A Cox proportional hazards model will be used for the final analysis of the primary outcome. Continuous parameters will be reported as the median and interquartile range (IQR) and compared with nonparametric tests. The Hodges-Lehmann estimate will be used to calculate 95% CIs for the difference between medians. Categorical variables will be reported as counts and percentages and compared with the χ2 test. We will measure Odds ratios for the clinical endpoint with the Mantel-Haenszel test. Kaplan-Meier test will be done to assess the time to clinical deterioration. We will use the log-rank test to compare endpoint–free survival between treatment and placebo group. Statistical significance will be set at P < .05, and all tests will be 2-tailed. IBM SPSS statistical software of the latest available version will be used for all statistical analyses. All reporting will adhere to the Consolidated Standards of Reporting Trials (CONSORT) guideline. The statistical analysis plans (SAP) providing all details of the analyses will be updated before conducting any data analysis.

Methods for additional analyses (e.g. subgroup analyses)

Additional analysis will be done for different gender and age subgroups. A Cox proportional hazards model will be used for the analysis of the primary endpoints of sub-groups. For analysis of the mortality of each of these subgroups, a logistic regression model will be used.

Methods in analysis to handle protocol non-adherence and any statistical methods to handle missing data

Multiple imputations will be used for missing data. The primary analysis will use the intention-to-treat principle and appropriate statistical analysis will be undertaken to assess the robustness of the findings

**Patient Safety Monitoring:**

Based on previous data the investigators anticipate colchicine to have an excellent safety profile given the medication's widespread use in the outpatient and inpatient setting. However outcome data at each 20 patients will be evaluated to ensure there are no adverse events which would necessitate pausing or discontinuation of the study. A signal for adverse events will be discussed internally with the investigators and reported to the IRB. Serious adverse events will be reported within 24 hours to the IRB.

Definition of Adverse Events (AE)-

‘A response to a drug which is noxious and unintended, and which occurs at doses normally used in man for the prophylaxis, diagnosis, or therapy of disease, or for the modifications of physiological function’. WHO, (1972)

Definition of Serious Adverse Events (SAE)-

Any untoward medical occurrence that at any dose;

–Results in death

–Life threatening

–Requires inpatient hospitalization or prolongation of existing hospitalization

–Results in persistent of significant disability or incapacity

**Study intervention discontinuation and participation withdrawal:**

Participant will preserve every right to discontinue treatment at any points of the study. This will not disqualify him or her right to get other standard treatment and care. All patient data will be protected from unauthorized access by others. Study physician will also preserve the right to discontinue study intervention if they justify that it will be better for the health of the participant.

**Ethical considerations:**

1. Ethical clearance for the study will be taken from the ERC of DMC.
2. Permission for the study was taken from the concerned department from where study subjects will be collected.
3. The entire study subject will be thoroughly appraised about the nature, purpose and implications of the study, as well as entire spectrum of benefits and risks of the study.
4. Interest of the study subjects will not be compromised to safeguard their rights and health.
5. All study subjects will be assured of adequate treatment of any complications developed in relation to the study purpose.
6. Study subjects will be assured about their confidentiality and freedom to withdrawn them from the study anytime.

**Budget and funding:**

Self-funding.

**Supporting documents and operational considerations:**

- Data collection form- Bangla and English
- SOPs

**References:**

1. Coronavirus disease (COVID-19) Situation Report–132Data as received by WHO from national authorities by 10:00 CEST, 31May 2020 (https://www.who.int/docs/default-source/coronaviruse/situation-reports/20200531-covid-19-sitrep-132.pdf?sfvrsn=d9c2eaef_2.)

2. FocosD ,Anderso A, Tang J,Tuccori M. 2020,Convalescent plasma therapy for Covid 19, Preprints([www.preprints.org](http://www.preprints.org))

3. Antonio V, Francesco F, Chiara P, Giovanni G, et al .Cytokine storm and colchicine potential role fighting SARS-CoV-2 pneumonia. Italian journal of medicine. Preprint. doi: 10.4081/itjm.2020.1284

4. Yvan J, Thomas H, et al., Should we stimulate or suppress immune responses in COVID-19? Cytokine and anti-cytokine interventions. Autoimmunity Reviews, <https://doi.org/10.1016/j.autrev.2020.102567>

5. WHO j Coronavirus disease (COVID-2019) R&D. [https://www.who.int/blueprint/priority-diseases/key-action/novel-coronavirus/en/. Accessed](https://www.who.int/blueprint/priority-diseases/key-action/novel-coronavirus/en/.%20Accessed) March 25, 2020.

6. Wang Y, Fan G, Salam A, et al. Comparative Effectiveness of Combined Favipiravir and Oseltamivir Therapy Versus Oseltamivir Monotherapy in Critically Ill Patients With Influenza Virus Infection. J Infect Dis. December 2019. <https://doi.org/10.1093/infdis/jiz656>.

7. Cao B, Wang Y, Wen D, et al. A Trial of Lopinavir-Ritonavir in Adults Hospitalized with Severe Covid-19. N Engl J Med. March 2020. <https://doi.org/> 10.1056/NEJMoa2001282.

8. National Guidelines on Clinical Management of Coronavirus Disease 2019 (COVID-19).Version 7.0 28 May, 2020, DGHS, MOHFW , Government of the People's Republic of Bangladesh

9. Sakpal TV. Sample size estimation in clinical trial. Perspect Clin Res. 2010; 1(2):67-9.

Appendix 1:

| **Mild illness (ILI)** | Patients with uncomplicated upper respiratory tract viral infection may have non specific symptoms such as fever, fatigue, cough (with or without sputum production), sore throat, nasal congestion, anorexia, malaise, or headache. Rarely, patients may also present with diarrhoea, nausea, and vomiting. The elderly and immunosuppressed may present with atypical symptoms. Symptoms due to physiologic adaptations of pregnancy or adverse pregnancy events, such as dyspnoea, fever, GI-symptoms or fatigue, may overlap with COVID-19 symptoms |
| --- | --- |
| **Pneumonia** | **Adult** with pneumonia but no signs of severe pneumonia and no need for supplemental oxygen. **Child** with non-severe pneumonia who has cough or difficulty breathing + fast breathing: fast breathing (in breaths/min): < 2 months: ≥ 60; 2–11 months: ≥ 50; 1–5 years: ≥ 40, and no signs of severe pneumonia |

**Clinical classification for case management :**

| 01 | Mild | Influenza like illness (ILI) |
| --- | --- | --- |
| 02 | **Moderate** | Pneumonia (CRB 65 score 0) |
| 03 | Severe | Severe Pneumonia, Sepsis (CRB 65 score 1 or more) |
| 04 | Critical | ARDS, Septic shock |

**CRB 65 Scoring system:**


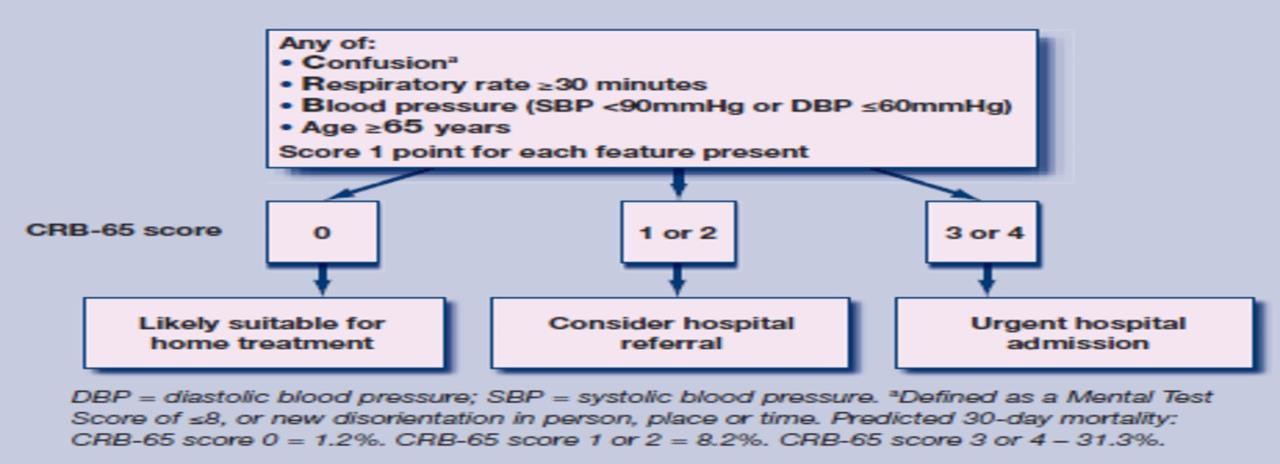


**Figure: CRB65 Scoring system**

**Definition of moderate COVID-19 disease: (must have all present)**

1. Fever or history of fever
2. Cough and /or Shortness of breath
3. Oxygen saturation 94% or more
4. Pneumonia –pulmonary consolidations on chest imaging (chest x ray or CT scan of chest) involving less than 50%of lungs
5. CRB 65 score 0
6. RT-PCR for SARS CoV-2 positive

**AewnZµ‡g m¤§wZcÎ**

**Title of Research: Colchicine in moderate symptomatic COVID-19 patients: double blind, randomized, placebo controlled trial to observe the efficacy**

**Principal Investigator:** Dr. Motlabur Rahman, Associate professor, Department of Medicine, Dhaka Medical College, Dhaka.

GB m¤§wZc‡Îi D‡Ïk¨ n‡jv Avcbv‡K cÖ‡qvRbxq Z_¨ cÖ`vb Kiv, †h Z_¨¸‡jv fv‡jvfv‡e †R‡bï‡b, ey‡S, cÖ‡qvR‡b cÖkœ K‡i Avcbv‡K wm×všÍ wb‡Z mvnvh¨ Ki‡e, Avcwb GB M‡elYvq AskMÖnY Ki‡eb wK bv?

**D‡Ïk¨ c×wZ:** Avgv‡`i GB M‡elYvwU **COVID-19** †ivMxi wPwKrmvi GKwU Ask wnmv‡e e¨envi Kiv n‡e| GB M‡elYvq †gwWwmb wefvM, XvKv ‡gwW‡Kj K‡jR nvmcvZv‡j ভর্তি রোগীদের অন্তর্ভুক্ত করা হবে। কিছুসংখ্যক রোগীকে দৈবচয়নের ভিত্তিতে Tablet colchicine- 0.6 mg নামক ঔষধ ১ ডোজ করে ১৪ দিন দেয়া হবে। অন্যদের প্রচলিত চিকিৎসা ও placebo দেয়া হবে। তাদেরকে নিয়মিত follow up করা হবে এবং ঔষধের কার্ষকারিতা পরীক্ষা করা হবে। আপনি কোন গ্রুপে অন্তর্ভুক্ত হবেন তা লটারিতে নির্ধারিত হবে।

**M‡elYvi SzuwK: colchicine নামক** ঔষধ খুবই সহনীয় ও উল্লেখযোগ্য ক্ষতিকর পাশ্বপ্রতিক্রিয়া নাই। Gic‡iI Avcbvi kix‡i mvgvb¨ cvk¦©cÖwZwµqv যেমন বমিভাব, পাতলা পায়খানা †`Lv w`‡Z cv‡i Z‡e Avgv‡`i cÖwk¶YcÖvß wPwKrmK GB ai‡bi cwiw¯’wZ wbqš¿‡Y m¶g|

**M‡elYvq AskMÖn‡Yi myweavw`:** GB M‡elYvq AskMÖnY Ki‡j Avcwb e¨w³MZfv‡e mivmwi jvfevb n‡Z cv‡ib| GB M‡elYv evsjv‡`‡k wPwKrmK‡`i GB †ivM m¤ú‡K© Av‡iv Rvb‡Z mnvqZv Ki‡e|

**weKí:** GB M‡elYvq AskMÖnY Kiv wKsev bv Kivi e¨vcv‡i ev AskMÖnY Kivi ci †h †Kvb mgq Avcwb wb‡R‡K M‡elYv †_‡K mwi‡q wb‡Z cv‡ib|

**LiP:** GB M‡elYvq AskMÖn‡Yi Rb¨ Avcbvi †Kvb LiP bvB ev Avcbv‡K †Kvb UvKv-cqmv †`qv n‡e bv|

**†MvcbxqZv:** M‡elYv PjvKvjxb I cieZ©x‡Z mKj Z_¨ K‡Vvifv‡e †Mvcb ivLv n‡e| cieZ©x‡Z d‡jvAvc I AbymiY cÖwµqvi Rb¨ Avcbv‡K GKwU AvBwW b¤^i †`Iqv n‡e| Avcbvi AvBwW b¤^i m¤^wjZ me ai‡bi KvMRc‡Î Avcbvi bvg I wVKvbv ewm‡q Awd‡mi dvBwjs †Kwe‡b‡U Zvjve× _vK‡e| e¨w³MZ welqvw` Z_¨ বিশ্লেষন, cÖwZ‡e`b ˆZwi‡Z Ges cÖKvkbvi Kv‡R e¨envi n‡e bv Ges M‡elYvi cix¶K e¨ZxZ Kv‡iv Kv‡Q cÖKvk Kiv n‡e bv| d‡j Avcbvi †Kvb Z_¨ Ab¨ †KD Rvb‡Z cvi‡e bv|

**†¯^”Qvg~jK AskMÖnY:** GB M‡elYvq Avcbvi AskMÖnY m¤ú~Y© †¯^”Qvg~jK| Avcwb M‡elYvq AskMÖn‡Y A¯^xK…wZ Rvbv‡Z cv‡ib A_ev M‡elYv PjvKvjxb †h †Kvb mgq M‡elYv †_‡K Avcbv‡K cÖZ¨vnvi K‡i wb‡Z cv‡ib| Zv‡Z Avcbvi wPwKrmvi †Kvb ZviZg¨ n‡e bv| GB di‡g ¯^v¶i Ki‡j Avcbvi AvBbMZ †Kvb AwaKvi Le© n‡e bv|

**cÖkœvejx:** hw` Avcbvi †Kvb cÖkœ _v‡K Z‡e `qv K‡i wRÁvmv Ki‡eb| Avgiv Zvi DËi cÖ`vb Kivi h_vmva¨ †Póv Ki‡ev| hw` fwel¨‡Z Avcbvi AwZwi³ †Kvb cÖkœ _v‡K Zvn‡j M‡elYviZ Wv³v‡ii mv‡_ †hvMv‡hvM Ki‡Z cv‡ib|

**m¤§wZi ¯^xKv‡ivw³:** Avwg M‡elYvq wb‡qvwRZ wPwKrmK-Gi mv‡_ (whwb Avgvi kvixwiK cix¶v Ki‡eb) GB M‡elYv wb‡q Av‡jvPbvq mš‘wó cÖKvk KiwQ| Avwg GUv ey‡SwQ †h M‡elYvq AskMÖnY †¯^”Qvg~jK Ges Avwg †h †Kvb mgq †Kvb eva¨evaKZv QvovB M‡elYv †_‡K Avgv‡K weiZ ivL‡Z cvwi| Avwg Dc‡iv³ kZ©¸‡jv c‡owQ/ Avgvi m¤§y‡L cwVZ n‡q‡Q Ges †¯^”Qvq M‡elYvq AskMÖnY Ki‡Z m¤§wZ Ávcb KiwQ|

mv¶vrKvi MÖnYKvixi ¯^v¶i: AskMÖnYKvixi ¯^v¶i:

ZvwiL: ZvwiL:

¯^v¶xi ¯^v¶i: 1)

2)

**INFORM WRITTEN CONSENT FORM**

**Title of Research: Colchicine in moderate symptomatic COVID-19 patients: double blind, randomized, placebo controlled trial to observe the efficacy**

**Principal Investigator:** Dr. Motlabur Rahman, Associate professor, Department of Medicine, Dhaka Medical College, Dhaka.

The purpose of this consent form is to give information so that you can take the decision on whether to take participation in this clinical trial or not after been informed properly.

**Purpose & nature of the study**: There is no specific antiviral agents for the treatment of COVID-19. In this work, we explore the efficacy of colchicine in moderate symptomatic patients to find out whether it is effective in preventing disease progression. After being involved in the study, you will be given a tablet named colchicine (0.6 mg) for 14 days along with standard treatment (Paracetamol, antihistamine, dexamethasone) if you randomized to treatment group. If you are randomized to control group, you will receive a placebo and standard treatment. Randomization will be done by computer generated randomization number.

**Expectation from and involvement of the participant:** During interview for enrollment you will be asked some question according to structured questionnaire that are all about your disease. It may take about half an hour to complete. We expect the information you are giving all will be correct. If you give consent blood and urine sample for test will be collected from you. Chest imaging will also be done. Some blood test will be repeated during follow up on day 3, 7 14, 21. You will be requested to take the prescribed medicine properly and participate in follow up process.

**Risks and benefit:** colchicine is generally well tolerated. There might be some side effects like nausea, diarrhea but our skilled specialists will manage these problems.

**Privacy, anonymity and confidentiality**: All information will be kept confidential and preserved by the researcher.

**Right to withdraw:** You are free to take part or refuse during any part of the study. Investigators will act as both investigator and physician. If you are agreeing to our proposal of enrolling you in our study, please indicate that by putting your signature or your left thumb impression at the specified space below.

Thank you for your co-operation.

Signature or left thumb impression Signature or left thumb impression

Of attendant / guardian /patient Of Witness

Signature of investigator

Date:

**Data Collection Sheet**

**Title of Research: Colchicine in moderate symptomatic COVID-19 patients: double blind, randomized, placebo controlled trial to observe the efficacy**

**Principal Investigator: Dr.** **Dr. Motlabur Rahman, Associate professor, Department of Medicine, Dhaka Medical College, Dhaka.**

**ID no: Date:**

1. Name of respondent:
2. Age (in years): years
3. Occupation:
4. Gender : i) Male ii) Female

|  |  |  |  |  |  |  |  |  |  |  |  |
| --- | --- | --- | --- | --- | --- | --- | --- | --- | --- | --- | --- |

1. Contact number:

6. Address:

8. Initial clinical feature:

i. Symptomatic, from (Date)

a) Fever

- - Date of first appearance of fever:
  - Pattern:
  - Highest recorded temperature:
  - Number of peaks in a day:

b) Cough

c) SOB

d) Sore throat

e) Diarrhoea

f) Others

9. Presence of Co-morbidity: i) No

ii) Yes: DM HTN Asthma/COPD CKD

Others

10. Patient is hospitalized in i. ward ii. HDU

11. Date of Admission:

12. Laboratory Data:

I. CBC: Hb TC NC LC PLT

II.RBS:

III.ALT:

IV.S. Creatinine:

V. CRP:

VI. Serum Ferritin:

13. Chest X-ray finding:

14. CT scan of chest finding:

15. Other treatment (along with total daily dose, starting and finishing date)

| **Name** | **Total daily dose** | **Starting** | **Finishing date** | **Days (Starting-finishing date)** |
| --- | --- | --- | --- | --- |
| Azithromycin |  |  |  |  |
| Hydroxychloroquine |  |  |  |  |
| Ivermectin |  |  |  |  |
| Doxycycline |  |  |  |  |
| Oral prednisolone |  |  |  |  |
| Methyl prednisolone |  |  |  |  |
| Enoxaparin |  |  |  |  |
| Favipiravir |  |  |  |  |
| Remdesivir |  |  |  |  |

16. Oxygen saturation (on day of randomization): , with room air

17. Maximum amount of oxygen supplement required ………………Lt/mi n,

Date: …………/……………/………………….

18. Patients highest category during hospital stay: (According to **seven-category ordinal scale**.)

[3] Hospitalized, not requiring supplemental oxygen;

[4] Hospitalized, requiring supplemental oxygen

[5] Hospitalized, requiring nasal high-flow oxygen therapy, noninvasive mechanical ventilation, or both

[6] Hospitalized, requiring ECMO, invasive mechanical ventilation, or both

[7] Death

19. in hospital outcome:

| i. Complete recovery  ii. Discharge with improvement  iii. Clinical deterioration: **( seven-category ordinal scale)**  4. hospitalized, requiring supplemental oxygen  5. hospitalized, requiring nasal high-flow oxygen therapy, noninvasive mechanical ventilation, or both  6. hospitalized, requiring ECMO, invasive mechanical ventilation, or both  iv. Death v. Treatment discontinued due to- |
| --- |

20. Outcome of patient at Day 21:

| 1. Clinical recovery  2. RT-PCR – NEGATIVE/POSITIVE/UNKNOWN  3. Still hospitalized  4. death  5. Lost to follow up |
| --- |

21. Date of complete resolution of fever:………………/……………………/……………………

22. Date of first RT PCR Positivity:


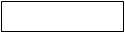


23. Date of 1^st^ RT PCR negativity:


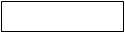


24. Date of 2^nd^ RT PCR negativity:


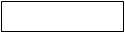


25. Date and Time of DEATH or DISCHARGE:


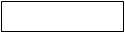


26. Follow up:

| **Day** | **Date** | **Highest Temperature till last f/u** | **Worst SpO_2_ till last f/u** | **seven-category ordinal scale** | **New treatment added including oxygen** | **CRP,**  **S. Ferritin,**  **Chest imaging** | **RT-PCR** |
| --- | --- | --- | --- | --- | --- | --- | --- |
| Day 3 |  |  |  |  |  |  |  |
| Day 7 |  |  |  |  |  |  |  |
| Day 10 |  |  |  |  |  |  |  |
| Day 14 |  |  |  |  |  |  |  |
| Day 21 |  |  |  |  |  |  |  |

Name and signature the of doctor

Date

**Screening for enrollment**

**Name of the participant: Age: …………..years**

**Title: Colchicine in moderate symptomatic COVID-19 patients: double blind, randomized, placebo controlled trial to observe the efficacy**

**Principal Investigator: Dr. Dr. Dr. Motlabur Rahman, Associate professor, Department of Medicine, Dhaka Medical College, Dhaka.**

1.

| **Inclusion criteria** |  |
| --- | --- |
| 1. Males and females of least 18 years of age and can swallow tablets |  |
| 1. Competent and willing to provide informed consent |  |
| 1. Patient must have received a diagnosis of COVID-19 infection with positive RT-PCR for SARS CoV-2 within the last 3 days |  |
| 1. Patients with moderate symptoms (According to National Guidelines on Clinical Management of Coronavirus Disease 2019 (COVID- 19).Version 7.0 .2020, DGHS, MOHFW, Government of the People's Republic of Bangladesh) |  |
| **Exclusion criteria** |  |
| 1. Pregnancy and breast-feeding |  |
| 1. Known hypersensitivity to colchicine |  |
| 1. Known chronic illness e.g hepatic failure, chronic kidney disease (eGFR<30ml/min), decompensated heart failure, long QT syndrome (QTc >450 msec.) |  |
| 1. Patient with inflammatory bowel disease (Crohn's disease or ulcerative colitis), chronic diarrhea or malabsorption |  |
| 1. Patient currently taking colchicine for other indications (mainly chronic indications represented by Familial Mediterranean Fever or gout) |  |
| 1. Patient undergoing chemotherapy for cancer |  |
| 1. Patient is considered by the investigator, for any reason, to be an unsuitable candidate for the study. |  |

2.

| **Selected** | **Not selected** |
| --- | --- |

3.

| **Assigned ID** |  |  |  |
| --- | --- | --- | --- |

NAME AND SIGN OF DOCTOR

DATE:

**Protocol amendment request**

**Trial Name**: **Colchicine in moderate symptomatic COVID-19 patients: double blind, randomized, placebo controlled trial to observe the efficacy**

**Trial No: 128**

We here by submitted few amendment for the ongoing trial “**Colchicine in moderate symptomatic COVID-19 patients: double blind, randomized, placebo controlled trial to observe the efficacy**” in the version 2 of the protocol.

1. Reformation of investigators list: the following investigators-

| Name | Role in the Study | Designations& place of posting | Signature |
| --- | --- | --- | --- |
| Prof. Khan Abul Kalam Azad | Study Supervisor and  Co-principal investigator | Principal , Dhaka Medical College , Dhaka , Bangladesh  Professor, Department of Medicine, DMC  Dean, post graduate medical faculty, university of Dhaka,  Councilor, BCPS. |  |
| Dr. Mahfuzul Haque | Co-Investigator | Assistant Professor,  Department of Medicine, Dhaka Medical College |  |
| Dr.Uzzwal Mallik | Co-Investigator | Assistant Professor,  Department of Medicine, Dhaka Medical College |  |
| Dr. Rifat H. Ratul | Co-Investigator | Medical officer, Department of Medicine, Dhaka Medical College, Dhaka, Bangladesh |  |

1. Renaming data collection form as “case record form”.
2. Removal of the patient identity data like address and phone number from case record form.
3. Addition in CRF: for more details of adverse outcome we want to add point 17. Adverse events:

i: Diarrhoea ,Duration …………………………..,Intervention:………………………………outcome…………………………….

ii: Abdominal pain/burning, Duration………………..., Intervention……………..……outcome…………………………….

iii: Nausea/ Vommitting, Duration ………………………..,Intervention:……………………outcome…………………………. iv: ………………………..,Duration …………………………..,Intervention:…………………………outcome…………………………

v: ………………………..,Duration …………………………..,Intervention:…………………………outcome…………………………

5. Addition of 28 day follow up: we want to extend one more follow up at day 28, so that the outcome at that point could be recorded- point 19. Outcome of patient at Day 28:

| 1. Clinical recovery  2. RT-PCR – NEGATIVE/POSITIVE/UNKNOWN  3. Still hospitalized- (score on seven category ordinal scale):  4. death  5. Lost to follow up |
| --- |

6. Addition of Table.1: Study timeline of participant assessments/interventions (supplied separately)

**Dr. Md. Motlabur rahman**

**Associate Professor, Department of Medicine and**

**Principal Investigator of the trial,**

**Dhaka Medical College, Dhaka, Bangladesh.**

**Office of the Ethical Review Committee**

**Dhaka Medical College Hospital**

**Dhaka, Bangladesh**

**Approval of Amendment**

**Ref-**Application from principal investigator of an ongoing trial “**Colchicine in moderate symptomatic COVID-19 patients: double blind, randomized, placebo controlled trial to observe the efficacy”.**

**Trial No-128**

The amendment proposed by the trial team in the second version of the trial protocol “**Colchicine in moderate symptomatic COVID-19 patients: double blind, randomized, placebo controlled trial to observe the efficacy”** was verified. It is believed that these change will improve the quality of the trial .So the ethical committee approve the following amendment in the version 2 of the trial protocol.

1. Addition of Co- Investigators List of the Protocol
2. Renaming data collection form as “case record form”.
3. Removal of the patient identity data like address and phone number from case record form.
4. Addition in CRF: more details of adverse outcome -point 17.
5. Addition of 28 day follow up to CRF- point 19.
6. Addition of Table.1: Study timeline of participant assessments/interventions

**Professor Dr. SM Shamsuzzaman**

**Head, Department of Microbiology and**

**Chairman, Ethical review committee and Data safety monitoring Board,**

**Dhaka Medical College**

**Dhaka, Bangladesh**
