# Supplementary material for: Efficacy of colchicine in patients with moderate COVID-19: A double-blinded, randomized, placebo-controlled trial
Source: PLoS One. 2022 Nov 16;17(11):e0277790. doi: 10.1371/journal.pone.0277790 (PMC9668149; doi:10.1371/journal.pone.0277790)
Supplement: Attachment — Submitted filename: Efficacy of Colchicine in moderate COVID.docx [file pone.0277790.s007.docx]

**Efficacy of Colchicine in moderate COVID-19 patients: A double-blind, randomized, placebo controlled trial**

**PONE-D-21-32054**

General comment

An important piece of work which is investigator-initiated and commendable. I have a couple of major and minor points for the consideration of the authors

**Major comments**

- **Role of Incepta Pharmaceuticals Ltd**

The authors make the point that the colchicine and the placebo was supplied by Incepta Pharmaceuticals Ltd. 40, Shahid Tajuddin Ahmed Srani, Tejgaon industrial area, Dhaka, Bangladesh …….. and the company had no role in planning, design, data collection, analysis …….

This turns out not to be exactly consistent with further comments. The authors report that the random numbers were generated and maintained by an independent biostatistician of Incepta pharmaceuticals Limited.

It the above is true, that represents substantial involvement in the drug producer in the design and implementation of the trial, and a case for conflict of interest.

- **How the arms were treated**

It is important that how patients in the arms of the study were treated be thoroughly detailed. Differences in care (beside the intervention) could account for the observed outcomes. Accordingly, I suggest that the distribution between the arms in terms of treatment as below is fully detailed in a table.

*Additionally, Paracetamol, antihistamines, oxygen therapy were also given as part of standard care according to the National guideline of Bangladesh and Clinical Management of COVID-19, Interim Guidance of World Health Organization [13][14]. Low molecular weight heparin, according to the indication, appropriate broad-spectrum antibiotics-if needed, Remdesivir injection as well as other drugs for associated comorbid conditions were also prescribed by the treating physicians.*

- **Blinding**

Colchicine has a very bitter taste. How did the investigator address this in the production of the placebos to preserve blinding?

- **Inclusion criteria and endpoint**

The study enrolled patients with oxygen concentration of no less than 94% without supplemental oxygen. How then did oxygen saturation greater than 93% without supplemental oxygen become an endpoint? That endpoint was achieved even at the time of enrolment.

- **Presentation**

All abbreviations in the tables should be written in full or have a key to guide readers.

- **Primary and secondary outcomes**

The sample size statement should make clear whether the assumptions are based on outcomes at Day 14 or 28.

The statement “Colchicine significantly reduced the **primary** outcome and all-cause mortality” is inaccurate as day 28 analysis was outcome of **secondary** analysis as per statements in the abstract & in line 242.

**Minor comments**

- State terms in full when they are first used e.g. Colchicine inhibits IL-1β, NLRP3 activation, ARDS, JAK ….…
- **C**olchicine need not be come with a capital **C**.
- **P**neumonia need not have a capital **P** in “World Health Organization (WHO) made notification of the **Pneumonia** case-cluster”
- **C**ytokine need not have a capital **C** in “The **Cytokine** storm syndrome….”
- It is unclear what benefit the untitled table in lines 183-4 add to the write-up. It can be well described in the text.
- It is possible to make the abstract less wordy e.g. “Data were collected using a case-record form.” Can be removed.
