# Supplementary material for: Efficacy of colchicine in patients with moderate COVID-19: A double-blinded, randomized, placebo-controlled trial
Source: PLoS One. 2022 Nov 16;17(11):e0277790. doi: 10.1371/journal.pone.0277790 (PMC9668149; doi:10.1371/journal.pone.0277790)
Supplement: Attachment — Submitted filename: Response to Reviewers.docx [file pone.0277790.s008.docx]

**A rebuttal letter**

To

Editor in chief,

PLOS ONE

**Subject: In response to review of the manuscript entitled “**Efficacy of colchicine in moderate COVID-19 patients: A double-blind, randomized, placebo controlled trial**”.**

Dear Sir,

Thank you for reviewing my manuscript. I have tried to address each point raised by the academic editor and the reviewers.

**Response to Academic editor**

1. PLOS ONE style-I have revised the manuscript according to PLOS ONE style.
2. The manuscript was edited by Editage. A copy was uploaded as supporting information. A clean copy of edited manuscript was uploaded as manuscript file.

**Review Comments to the Author**

Please use the space provided to explain your answers to the questions above. You may also include additional comments for the author, including concerns about dual publication, research ethics, or publication ethics. (Please upload your review as an attachment if it exceeds 20,000 characters)

Reviewer #1: This is an interesting RCT done in Bangladesh to evaluate the efficacy of colchicine in reducing Covid-19 severity in hospitalized patients. So far, the summary of the published evidence points to colchicine having no influence on mortality or progression in people hospitalized with moderate or severe Covid, and colchicine having an uncertain effect on people with mild or asymptomatic diseases. Therefore, more evidence is welcome.

My main concern for this study is that the severity of Covid-19 patients on enrolment is not very clear, as despite including only patients requiring hospitalization, less than 5% of patients needed oxygen in both arms during hospital stay. So this is quite unusual unless they were all hospitalized based on other-medical reasons, these participants behave like an outpatient setting. Furthermore, about 60% of patients received dexamethasone, which would have been incorrectly prescribed if over 95% of patients did not require oxygen. Also, the sample size calculation does not seem appropriate as further detailed below.

**Author’s response:** Thank you. It is true that these participants behave like an out patients setting. We enquired into the causes of their hospitalization and found the following reasons-

1) Among the participants, 148 (50%) patients had at least one of the comorbid conditions like diabetes mellitus, hypertension, COPD/ bronchial asthma, chronic kidney disease, chronic liver disease, ischemic heart disease.

2) Along with the moderate COVID-19 disease some patients also admitted either for the management of the coexisting disease like rheumatoid arthritis, SLE, epilepsy, myelo-proliferative disease or for the evaluation of co-existing clinical feature like hepatomegaly, central chest pain, lower limb weakness, abdominal pain (later diagnosed as cholecystitis), pancytopenia.

3) More than 11% of our study participants are from the age group of 61 years or more. Considering their risk of development of the severe disease these patients were admitted during the initial phase of the pandemic.

4) Some patients with moderate COVID-19 were also hospitalized due to the severity of their symptoms like intense and irritating cough, high fever for the duration of 7 or more days, fever not responding to paracetamol. 8.8% of the patients presented with diarrhea with variable degree of dehydration which leads to their hospitalization.

5) Less than 5% of our participants are health care workers, despite having moderate COVID-19 they were hospitalized as part of providing institutionalized isolation facilities.

Concern regarding less than 5% of patients needed oxygen- during 14th day follow up we found only 14 (on oxygen) plus 7 (death-also required oxygen) patients i,e 21 patients (7.2%) required oxygen. When we analyzed the highest in hospital severity in terms of 7 category ordinal scale, we found that 68 (23%) patients required supplemental oxygen at least for few hours during their hospital stay.

Concern about 60% of patients received dexamethasone- about 23% of the patients needed supplemental oxygen in our study but about 60% received dexamethasone. We appreciate your concern and agree with you that the use of dexamethasone might be inappropriate during the initial period of the trial. We started patient enrolment from 15^th^ July, 2020 and WHO provide the guidance regarding dexamethasone use during the first week of September, 2020. During the initial phase of the pandemic there was a tendency to use dexamethasone based on few preprint reports with the intention to help patients with whatever evidence we have.

INTRODUCTION.
• The authors state that there is no effective treatment for Covid-19 inflammatory response; however, corticosteroids have been shown to reduce mortality in COVID pneumonia (Recovery trial)

**Author’s response:** thank you. Information is added in the manuscript. (Line-112)

Also, there are several systematic reviews on Covid-19 and colchicine. The most comprehensive and rigorous is a recent COCHRANE review (doi: 10.1002/14651858.CD015045) evaluating colchicine for the treatment of Covid-19. It included three RCTs with over 11 thousand hospitalized participants and concluded that based on the current evidence, in people hospitalized with moderate to severe COVID-19 the use of colchicine probably has little to no influence on mortality or clinical progression. However, there is uncertain evidence of the effect of colchicine on mortality for people with asymptomatic infection or mild disease and colchicine probably results in a slight reduction of hospital admissions or deaths within 28 days, and the rate of serious adverse events compared with placebo. These results from the systematic review should be included in the manuscript.

**Author’s response:** Thank you. Information from COCHRANE review is added in the manuscript. (Line-123-132)

METHODS
• Page 16 of Study Protocol in supplementary information states, "All patients will be in category 3, hospitalized, not requiring supplemental oxygen; according to seven-category ordinal scale. Patients with other categories will not be included in this study”. This should also be mentioned equally clearly in the main paper.

**Author’s response:** Thank you. This is added under ‘patients and methods’ heading from line 180 to 182.

• It is not clear if radiologic pneumonia was a required criterion for enrolment or not. This is not mentioned in the paper, although it was included as an inclusion criterion in clinical.trials registry.

**Author’s response:** thank you. This is added under ‘patients’ heading from line 173 to 174.

Concerns regarding sample size calculation:
 The primary outcome was “time to develop clinical deterioration”, defined as the time from randomization to a deterioration of two or more points (from the status at randomization) on a seven-category ordinal scale. However, sample size calculation was done based on the "proportion of subjects that would be cured by colchicine”. Therefore, sample size calculation does not seem to have been calculated on the same outcome than the primary outcome.
o Additionally, the authors provide no reference for the “assumption that the proportion of subjects that would be cured by colchicine is 50%” nor for the estimated “16%” difference for the outcome between both study arms.

**Author’s response:** Thank you. Necessary correction has been made in the respective section. (Line-275-291)

RESULTS:
*The proportion of pneumonia in chest images in each group is not shown, although all participants had a chest image done per protocol.*

**Author’s response:** Thank you. Proportion of pneumonia in chest images was not recorded in the case record form. We enrolled the patients if CT chest involvement is less than 50%. In case of chest x-ray, patients were enrolled only if at least two of the investigators agreed that lung field involvement is less than 50%, as percentage of involvement in chest x-ray is not routinely reported by radiologist in our settings.

*Remdesevir use was allowed per-protocol but is not reported in table 1*

**Author’s response:** Thank you. This is added in table 1.

• There is an error in the calculation for the proportion of participants having received dexamethasone in both study arms and reported in Table 1: colchicine arm 97/148 = 65.5% (and not 32.8%) and in control arm 85/148 =57.4% (and not 28.7%). The same miscalculation is found for the LMWH proportion of use in both arms in Table 1.

**Author’s response:** Thank you. This is corrected in table 1.

*It is striking that in a clinical trial of patients hospitalized for Covid-19, less than 5% (14 of 292 as shown in Table 2) of all participants required supplemental oxygen, and less than 4.5 % deteriorated (primary outcome), considering that mean day of hospital stay was 11 days.
Also, if sample size calculation assumption was that 50% would deteriorate in control arm, this assumption was very far from what really happened and the estimation was not met.*

**Author’s response:** Thank you. During 14th day follow up we found only 14 (on oxygen) plus 7 (death-also required oxygen) patients i,e 21 patients (7.2%) required oxygen. When we analyzed the highest in hospital severity in terms of 7 category ordinal scale, we found that 68 (23%) patients required supplemental oxygen at least for few hours during their hospital stay.

Assumption about 50% deterioration to scale 5 or more in the 7-category ordinal scale in control arm was made considering the presence of comorbidities and the age of the patients which are responsible for the hospitalization of the moderate COVID-19 patients. Also, the course and the prognosis were not established like now a day. However, we agree with you that the assumption was far from the reality.

*Three deaths occurred after hospital discharge (1 in colchicine arm, two in control arm) and no information is provided.*

**Author’s response:** Thank you. As they died at home their cause of death could not be established but information is added from line 424 to 435.

• It is unclear why the authors did not do a multivariate analysis.

**Author’s response:** thank you. Multivariate analysis was done but the authors did not include them in the manuscript as they thought it would add very little new information.

• English writing could be improved.

**Author’s response:** Thank you. We have edited our writing.

Reviewer #2: Efficacy of Colchicine in moderate COVID-19 patients: A double-blind, randomized, placebo-controlled trial
PONE-D-21-32054
General comment
An important piece of work which is investigator-initiated and commendable. I have a couple of major and minor points for the consideration of the authors
Major comments
*• Role of Incepta Pharmaceuticals Ltd
The authors make the point that the colchicine and the placebo was supplied by Incepta Pharmaceuticals Ltd. 40, Shahid Tajuddin Ahmed Srani, Tejgaon industrial area, Dhaka, Bangladesh …….. and the company had no role in planning, design, data collection, analysis …….
This turns out not to be exactly consistent with further comments. The authors report that the random numbers were generated and maintained by an independent biostatistician of Incepta pharmaceuticals Limited.
It the above is true, that represents substantial involvement in the drug producer in the design and implementation of the trial, and a case for conflict of interest.*

**Author’s response:** thank you. Necessary corrections made in line 162-164 and ‘conflict of interest’ section.

*• How the arms were treated
It is important that how patients in the arms of the study were treated be thoroughly detailed. Differences in care (beside the intervention) could account for the observed outcomes. Accordingly, I suggest that the distribution between the arms in terms of treatment as below is fully detailed in a table.
Additionally, Paracetamol, antihistamines, oxygen therapy were also given as part of standard care according to the National guideline of Bangladesh and Clinical Management of COVID-19, Interim Guidance of World Health Organization [13][14]. Low molecular weight heparin, according to the indication, appropriate broad-spectrum antibiotics-if needed, Remdesivir injection as well as other drugs for associated comorbid conditions were also prescribed by the treating physicians.*

**Author’s response:** thank you. Distribution of Remdesivir, injectable antibiotics like ceftriaxone, meropenem is added in table 1.

*• Blinding
Colchicine has a very bitter taste. How did the investigator address this in the production of the placebos to preserve blinding?*

**Author’s response:** thank you. Only excipients were used to produce the placebo. Nothing was added to make the placebo bitter. We advised the patients to take the tablet with water and to ingest before melting in mouth.

*• Inclusion criteria and endpoint
The study enrolled patients with oxygen concentration of no less than 94% without supplemental oxygen. How then did oxygen saturation greater than 93% without supplemental oxygen become an endpoint? That endpoint was achieved even at the time of enrolment.*

**Author’s response:** thank you. ‘Oxygen saturation greater than 93% without supplemental oxygen’ was not an endpoint.

*• Presentation
All abbreviations in the tables should be written in full or have a key to guide readers.*

**Author’s response:** thank you. Abbreviations are written in full below the table.

• Primary and secondary outcomes
The sample size statement should make clear whether the assumptions are based on outcomes at Day 14 or 28.
The statement “Colchicine significantly reduced the primary outcome and all-cause mortality” is inaccurate as day 28 analysis was outcome of secondary analysis as per statements in the abstract & in line 242.

**Author’s response:** thank you. Necessary correction has been made in the manuscript.

*Minor comments
• State terms in full when they are first used e.g. Colchicine inhibits IL-1β, NLRP3 activation, ARDS, JAK ….…*

**Author’s response:** thank you. Terms are stated in full at their first appearance.

• *Colchicine need not be come with a capital C.
• Pneumonia need not have a capital P in “World Health Organization (WHO) made notification of the Pneumonia case-cluster”
• Cytokine need not have a capital C in “The Cytokine storm syndrome….”
• It is unclear what benefit the untitled table in lines 183-4 add to the write-up. It can be well described in the text.
• It is possible to make the abstract less wordy e.g. “Data were collected using a case-record form.” Can be removed*.

**Author’s response:** thank you. All suggested correction has been made in the manuscript.

Reviewer #3: A randomized clinical trial was conducted which aimed to determine whether adding colchicine with other standard of care treatment improved moderate COVID-19 pneumonia. A late beneficial effect of colchicine was observed compared to standard of care.

Minor revisions:
1- Line 250: Is “cured” the correct terminology?
2- Line 248: Indicate the statistical testing method which achieves 80% power.
3- Line 257: Perhaps the more precise terminology is “dependent” instead of “related.”
4- Line 273: Remove the comma between December and 15.
5- Table 1: Clarify the results reported for the placebo group’s duration of symptoms.
6- Line 325: Replace “across” with “between” since there are only two groups.
7- Indicate if adverse events were collected according to a standardized method, such as CTCAE.
8- Figure 2: To more clearly display the time to event data, cut the y-axis at 0.25.
9- Figure 3: (a) Provide a more descriptive y-axis label. (b) What days do the number at risk correspond to? The layout makes this difficult to determine.
10- Thoroughly proofread the manuscript.

**Author’s response to minor revisions:** thank you for nice suggestions. Corrections have been made accordingly.

I hope I have tried my level best to address all of your point raised during the review process. Please consider my manuscript for publication in PLOS ONE.

Thanks

**Dr. Ponkaj Kanti Datta**

Assistant professor

Department of medicine

Dhaka Medical College
