# Supplementary material for: Efficacy of colchicine in patients with moderate COVID-19: A double-blinded, randomized, placebo-controlled trial
Source: PLoS One. 2022 Nov 16;17(11):e0277790. doi: 10.1371/journal.pone.0277790 (PMC9668149; doi:10.1371/journal.pone.0277790)
Supplement: Attachment — Submitted filename: Response to Reviewers.docx [file pone.0277790.s009.docx]

**A rebuttal letter**

To

Editor in chief,

PLOS ONE

**Subject: In response to review of the manuscript entitled “**Efficacy of colchicine in moderate COVID-19 patients: A double-blind, randomized, placebo controlled trial**”.**

Dear Sir,

Thank you for reviewing my manuscript. I have tried to address each point raised by the academic editor and the reviewers.

**Response to Academic editor:**

1. PLOS ONE style-I have revised the manuscript according to PLOS ONE style.
2. The manuscript was edited by Editage. A copy was uploaded as supporting information. A clean copy of edited manuscript was uploaded as manuscript file.

Additional Editor Comments:
The reviewers are mostly positive about your revised manuscript, but have raised remaining concerns that need to be addressed in a revision, in particular please consider Reviewer#1 comments.

**Response to the editor:**

**Thank you very much. We have tried to address the concerns raised by the respected reviewer’s.**

[Note: HTML markup is below. Please do not edit.]

Reviewers' comments:

Reviewer's Responses to Questions

**Comments to the Author**

1. If the authors have adequately addressed your comments raised in a previous round of review and you feel that this manuscript is now acceptable for publication, you may indicate that here to bypass the “Comments to the Author” section, enter your conflict of interest statement in the “Confidential to Editor” section, and submit your "Accept" recommendation.

Reviewer #1: (No Response)

Reviewer #2: All comments have been addressed

Reviewer #3: (No Response)

2. Is the manuscript technically sound, and do the data support the conclusions?

The manuscript must describe a technically sound piece of scientific research with data that supports the conclusions. Experiments must have been conducted rigorously, with appropriate controls, replication, and sample sizes. The conclusions must be drawn appropriately based on the data presented.

Reviewer #1: Partly

Reviewer #2: Yes

Reviewer #3: Yes

3. Has the statistical analysis been performed appropriately and rigorously?

Reviewer #1: Yes

Reviewer #2: Yes

Reviewer #3: Yes

4. Have the authors made all data underlying the findings in their manuscript fully available?

The [PLOS Data policy](http://www.plosone.org/static/policies.action#sharing) requires authors to make all data underlying the findings described in their manuscript fully available without restriction, with rare exception (please refer to the Data Availability Statement in the manuscript PDF file). The data should be provided as part of the manuscript or its supporting information, or deposited to a public repository. For example, in addition to summary statistics, the data points behind means, medians and variance measures should be available. If there are restrictions on publicly sharing data—e.g. participant privacy or use of data from a third party—those must be specified.

Reviewer #1: Yes

Reviewer #2: Yes

Reviewer #3: Yes

5. Is the manuscript presented in an intelligible fashion and written in standard English?

PLOS ONE does not copyedit accepted manuscripts, so the language in submitted articles must be clear, correct, and unambiguous. Any typographical or grammatical errors should be corrected at revision, so please note any specific errors here.

Reviewer #1: Yes

Reviewer #2: Yes

Reviewer #3: Yes

6. Review Comments to the Author

Please use the space provided to explain your answers to the questions above. You may also include additional comments for the author, including concerns about dual publication, research ethics, or publication ethics. (Please upload your review as an attachment if it exceeds 20,000 characters)

Reviewer #1: Major comments:
1. Outcomes at 28 days were not included in Primary nor in Secondary study outcomes as defined in Methods section line 224-225 (“The time frame for all outcomes was 14 days post-randomization”). Therefore additional outcomes at 28 days should be considered exploratory and not “primary outcomes”. Despite this issue was already raised by Reviewer nº2, the manuscripts state in several paragraphs that on day 28, colchicine “significantly reduced the primary outcome” and all-cause mortality. This must be carefully corrected in the Abstract (lines 65, 68), Results section (lines 332, 334, 336) and in the Discussion (line 381) section.

Author’s response: thank you very much. The author’s are agreed with you. We are sorry for missing to respond properly to the previous reviewer. We have made necessary corrections in the above mentioned lines.

2. I have commented in my previous revision this: “The proportion of pneumonia in chest images in each group is not shown, although all participants had a chest image done per protocol.”
The authors responded: “Proportion of pneumonia in chest images was not recorded in the case record form. We enrolled the patients if CT chest involvement is less than 50%. In case of chest x-ray, patients were enrolled only if at least two of the investigators agreed that lung field involvement is less than 50%, as percentage of involvement in chest x-ray is not routinely reported by radiologist in our settings.”
I am sorry that my question wasn't clear enough. What I wanted to say is that it is important that the authors show how many participants in each study arm did have radiologic pneumonia on admission (the proportion of participants having pneumonia in each group), not the area or amount of radiologic involvement. This information is important for the readers, and if available, it should be included in Table 1.

Author’s response: thank you. According to inclusion criteria we included the patients with moderate symptomatic pneumonia. According to National Guidelines on Clinical Management of Coronavirus Disease 2019 (COVID- 19),Version 7.0 .2020, directorate general of health services (DGHS), ministry of health and family welfare (MOHFW), Government of the People's Republic of Bangladesh, moderate pneumonia patients must have radiologic pneumonia but the involvement would be less than 50% (refer to protocol –page 24). So, all the enrolled patients of this study had radiologic pneumonia. We are sorry that, it was not clear in our previous manuscript. We added “(*i.e. all the patients of both arms had radiologic pneumonia during enrolment*)” at line 149 in “patients” section for further clarification.

Minor comments:
3. Line 93, add “inhibitors” after IL-1

Author’s response: thank you. Added

4. Line 113: remove the extra period after COVID-19

Author’s response: thank you. Removed

5. Line 153-155: “All patients enrolled in this study were assigned to category 3, were hospitalized, and did not require supplemental oxygen, according to a seven-category ordinal scale.” The readers do not know at that point of the manuscript to which category the authors are referring until much later in the manuscript. I suggest to add a reference to WHO categories or refering in brackets something as “as defined in the Outcomes Measures section”.

Author’s response: thank you for nice suggestion. “as defined in the Outcome Measures section” was added in the manuscript.

6. Line 169 (last line of the Blinding Table) states that “statistician” were not blind. Were these statisticians also involved in data results analysis? What was the role of this “unblind” statisticians? This is confusing

Author’s response: thank you. The confusing part is removed. They were not involved in data result analysis.

7. The authors explained in their response to reviewers that the placebo had the same excipients than colchicine pills, but in Line 184 the authors state that “placebo tasted similar to the study drugs”. Having the same excipients does not necessarily give the same bitter taste.

Author’s response: thank you. We do agree with you. The authors admitted that the bitter taste of colchicine could have compromised the effort to blind. Necessary changes were made at Line185-188 and 426-428.

8. Line 264, add the year 2020 after December 15.

Author’s response: added

9. Table 1 shows results for 296 participants but 299 were randomized, this difference should be explained in the results section, line 265

Author’s response: thank you. The explanation is added at Line 268-270.

10. Table 1, under comorbidities, several abbreviations, such as CLD, CKD, COPD are undefined. This was already mentioned by Reviewer nº2.

Author’s response: thank you. Correction is done.

11. Was body mass index, or obesity measured in both groups? This is an important risk factor for severe COVID-19

Author’s response: thank you. Authors agree with you that body mass index (BMI) is an important risk factor but unfortunately there is no record of BMI in our case record form.

12. Line 332, replace P-0.0035 with p=0.0035

Author’s response: thank you. Replaced

13. Line 331, remove upper-case from “Colchicine” and “Hazard” ratio

Author’s response: thank you. Removed

14. Line 335 “ALT” is not previously defined

Author’s response: thank you. Edited and defined when it first appeared at Table 1.

15. Line 355-358 the description regarding liver testing abnormalities in both study arms is a little confusing, I suggest revising the writing

Author’s response: thank you. Edited at line 362 to 369.

16. Discussion, line 379-380. The authors state that “colchicine-treated patients deteriorated 1 day later than the placebo group”. This finding was not statistically significant, therefore it should be removed.

Author’s response: thank you. The line is removed.

17. Discussion: Line 383-389, revise text wording, several sentences are not well written

Author’s response: thank you. This part has been revised for word and language by Editage *(www.editage.com)*.

18. In general, the Discussion could be revised and much improved. For example, in line 396-402 all the discussion about CRP levels is unclear, I suggest revising carefully. How can the authors conclude that the CRP decrease relates to anti-inflammatory drug use and not just on clinical natural recovery of patients? This looks speculative.

Author’s response: thank you. Corrections have been made according to your suggestion (line 410-418).

19. Any further discussion on the limitation of having over 2/3 of participants on both arms having received dexamethasone? Do the authors think that colchicine may have an additive beneficial effect? (this is where a multivariate analysis may have helped in the results interpretation…)

Author’s response: thank you. The authors agreed not to add further discussion.

Reviewer #2: The authors have done well to address the comments made by reviewers. I believe the manuscript is a stage where readers will be able to judge well its merits and demerits. Regarding blinding, I will recommend to the authors to consider admitting that the bitter taste of colchicine could have compromised the effort to blind.

Author’s response: thank you for your nice recommendation. The authors admitted that the bitter taste of colchicine could have compromised the effort to blind. Necessary changes were made at Line184-186 and 411-413.

Reviewer #3: Minor Revision:
Figure 3: What time points do the number at risk correspond to? The layout makes this difficult to determine. Placing the number at risk immediately below the Number of Days Survived on the x-axis may clarify.

Author’s response: thank you. It is corrected according to your suggestion.

I hope I have tried my level best to address all of your concerns raised during the review process. Please consider my manuscript for publication in PLOS ONE.

Thanks

**Dr. Ponkaj Kanti Datta**

Assistant professor

Department of medicine

Dhaka Medical College
