# Supplementary material for: Efficacy of colchicine in patients with moderate COVID-19: A double-blinded, randomized, placebo-controlled trial
Source: PLoS One. 2022 Nov 16;17(11):e0277790. doi: 10.1371/journal.pone.0277790 (PMC9668149; doi:10.1371/journal.pone.0277790)
Supplement: Attachment — Submitted filename: Response to Reviewers.docx [file pone.0277790.s010.docx]

**A rebuttal letter**

To

Editor in chief,

PLOS ONE

**Subject: In response to review of the manuscript entitled “**Efficacy of colchicine in moderate COVID-19 patients: A double-blind, randomized, placebo controlled trial**”.**

Dear Sir,

Thank you for accepting our response to the concerns raised by the reviewer’s. We have copyedited the newly added/corrected text for language usage, spelling, and grammar by Editage as you recommended. I have reviewed the reference list carefully and checked the figure files with PACE to ensure that they meet PLOS requirements.

I hope I have tried my level best to address all of your concerns raised during the review process. Please consider my manuscript for publication in PLOS ONE.

Thanks

**Dr. Ponkaj Kanti Datta**

Assistant professor

Department of medicine

Dhaka Medical College
